# Cytoskeleton Markers in the Spinal Cord and Mechanoreceptors of Thick-Toed Geckos after Prolonged Space Flights

**DOI:** 10.3390/life12010100

**Published:** 2022-01-11

**Authors:** Alexandra Proshchina, Victoria Gulimova, Anastasia Kharlamova, Yuliya Krivova, Valeriy Barabanov, Sergey Saveliev

**Affiliations:** A.P. Avtsyn Research Institute of Human Morphology, Ministry of Science and Higher Education RF, Tsurupi Street, 3, 117418 Moscow, Russia; gulimova@yandex.ru (V.G.); grossulyar@yandex.ru (A.K.); homulkina@rambler.ru (Y.K.); barabanovgrant@mail.ru (V.B.); embrains@mail.ru (S.S.)

**Keywords:** space flight, hypogravitational motor syndrome, cytoskeleton, thick-toed geckos, mechanoreceptors, spinal cord, motor neurons, glia, ‘Bion-M1’ biosatellite, ‘Foton-M3’ satellite

## Abstract

Spaceflight may cause hypogravitational motor syndrome (HMS). However, the role of the nervous system in the formation of HMS remains poorly understood. The aim of this study was to estimate the effects of space flights on the cytoskeleton of the neuronal and glial cells in the spinal cord and mechanoreceptors in the toes of thick-toed geckos (*Chondrodactylus turneri* GRAY, 1864). Thick-toed geckos are able to maintain attachment and natural locomotion in weightlessness. Different types of mechanoreceptors have been described in the toes of geckos. After flight, neurofilament 200 immunoreactivity in mechanoreceptors was lower than in control. In some motor neurons of flight geckos, nonspecific pathomorphological changes were observed, but they were also detected in the control. No signs of gliosis were detected after spaceflight. Cytoskeleton markers adequately reflect changes in the cells of the nervous system. We suggest that geckos’ adhesion is controlled by the nervous system. Our study revealed no significant disturbances in the morphology of the spinal cord after the prolonged space flight, supporting the hypothesis that geckos compensate the alterations, characteristic for other mammals in weightlessness, by tactile stimulation.

## 1. Introduction

The development of life on Earth took place under the influence of the forces of gravity. Under conditions of space flight, the loads on the musculoskeletal system and vestibular afferentation are changed, and the adaptation of the central nervous system to new conditions is needed. Respectively, the coordination of signals coming from the musculoskeletal system and vestibular organs are restructured [1,2]. In conditions of weightlessness, a range of physiological alterations and symptoms (headward fluid shifts; headaches; back pain; and changes in the cardiovascular, bone, and muscle systems) are developed in human and experimental animals [3]. They are collectively referred to as space adaptation syndrome (SAS).

Hypogravitatational motor syndrome (HMS) is one of the most remarkable manifestations of SAS and one of the deleterious impacts of weightlessness on the body in orbital space missions [4]. HMS is developed in conditions of cancellation or significant reduction of gravitational loads. It is characterised by the deep disturbances of the main proprioceptive activity—vestibular, muscular, supporting systems—including specific changes in skeletal muscles, particularly in the so-called postural muscles responsible for maintaining posture in the gravity field of the Earth [4,5,6,7,8] and alterations in functional and structural characteristics of the skeletal muscles—atony, atrophy, decreased strength contractions, and increased muscle fatigue [9].

Earlier, it has been shown that the important trigger of HMS is violation of sensory impulses from the skin [5]. For example, the absence of sensory stimuli from mechanoreceptors of the sole skin possibly results in the functional activity of motor neurons innervating leg muscles [8]. Neuro- and molecular-physiological study results suppose the supporting system afferentation acting as a trigger in tonic regulation system is a key factor in HMS [7,10]. However, it is likely there are no special gravity receptors. Gravity is detected via many types of sensory receptors that project a sense of acceleration and/or gravitational loading. For example, different types of receptors from the inner ear detect acceleration and gravitational loading, allowing the nervous system to accommodate and control motor answer to gravitational force. The presence of various combinations and types of input from different combinations of receptors enables motor control systems to accommodate and to respond to gravitational forces [11].

It is also suggested that many of these ensembles are responded to by spinal cord neurons, which can, in turn, realise detailed accommodation of posture and locomotion [11]. Moreover, there is a hypothesis that disorders of the musculoskeletal system as part of HMS arise in consequence of changes in spinal motor neurons [4,12]. Functionally and topographically different motor neurons and the muscles innervated by them respond differently to space flight factors. Analysis of the HMS development dynamics supports this statement. In the skeletal muscles, which maintain the posture, the pathological shifts develop during the first day [7,9,13]. Within the first few hours of the gravitational unloading, muscular tone of the extensors collapses (up to 40–50%) [7]. Simultaneously, a decrease of their force and endurance and suppression of postural tremor are registered [13]. Pathognomonic signs in the skeletal muscles are also shown after peripheral nerve injury [12].

For decades, it has been shown that acute changes in gravity have an effect on neuronal systems of human and animals on different levels, from the molecular level to the whole nervous system [14,15,16,17]. This aspect of HMS development is still poorly explored [13]. However, some reports on the spinal cord of rodents after orbital flights are available.

In weightlessness, a decreased activity was indicated for spinal ganglia neurons and motoneurons of the spinal cord [14]. In rat spinal motor neurons, the content of cytoplasmic proteins was significantly lowered [18]. After 30 days of space flight, in motor neurons of mice lumbar spinal cords, immunoexpression of the proteins responsible for synaptic transfer of a nervous impulse and proteins of heat shock proteins decreased [19]. In general, these results support the assumption that weightlessness affects the motor neurons of the spinal cord, which is a leading factor in HMS [8].

In the antiorthostatic head-down rest experiment (hypogravitational models—hind limb unloading model (HUM)) [20,21], decreasing of the neurotransmitters (GABA, glycine, glutamate, aspartate) was detected in the rat cord in the fifth lumbar vertebra, which contains motor neurons innervating *musculus Soleus*. No changes were observed in the fourth lumbar vertebra, whose motoneurons maintain the *musculus Tibialis anterior* and *musculus Plantaris* [13].

Lumbar enlargement was shown to decrease after 35 days of antiorthostatic head-down rest experiment, while the square of the transversal serial sections of the cervical enlargement remained unchanged [22]. This decrease was a result of the white matter reduction. Glial/nervous cell death was hypothesised as the cause, due to the data that showed hypogravitational modelling leads to an increased probability of cells of different types entering apoptosis. At the same time, the effect of weightlessness on the white matter fibres and neuroglial cells remains unstudied. Glial alterations are thought may to be a source for the spinal cord aberration [13]. In the HUM model, an increase of the certain proteins, relating to astro- and oligodendroglial cells (S100B, HSP25, HSP70, Olig2), was shown. Molecular analysis revealed a decrease of *pmp2* and *pmp22* gene expression coding myelin, which led to demyelination effects, destruction of the myelin sheaths, and axon conduction velocity changes. Moreover, signs of demyelination and decreasing oligodendroglial cells have been shown in mice after 30 days of exposure to microgravity. However, the HUM study revealed no changes in motoneuron apoptotic level or alterations in the quantity of the myelinated nerve fibres in the anterior nerve roots [9]. The data on the potential spinal cord demyelination under space flight conditions could supplement explanation of the HMS pathogenesis.

To test the hypothesis that changes in spinal motor neurons provoke HMS, researchers performed a full genome study of the mice lumbar spinal cord after the 30-day ‘Bion-M1’ mission [23]. Data published to date generally provide evidence for the spinal cord motoneuron functional plasticity under altered gravitational forces. In the transcriptional profile analysis of the mice spinal cord after 30 days of space flight and readaptation, 178 genes, involved in 60 metabolic and signal pathways, with changed expression, were detected. A total of 118 genes of the increased expression and 60 of the decreased expression were revealed [8]. In the study of Islamov et al. [23], cytoskeletal proteins were shown to participate in spinal cord alteration.

Cytoskeleton elements, including microtubules, intermediate filaments, and microfilaments, were shown to reorganise in the condition of microgravity [24,25]. Changes in cell architecture led to alteration of cell response to the environment. Cytoskeletal rearrangements occur just a few minutes after exposure to altered gravitation [26]. These changes differ between types of cells [24,27,28,29]. Actin and tubulin filaments undergo such alterations most frequently [30]. Transitional rearrangements of the cytoskeleton elements and associated membrane receptors are supported to be the first step of adaptation to microgravity due to their quick initiation [26]. Microtubule alterations were also observed for rat glial cells [25] under the experimental clinostat microgravity. These cytoskeleton changes could be involved in neurophysiological disturbances, which have been observed during spaceflight.

In general, the best known gravisensitive proteins are cytoskeletal proteins (for review, see [31]). However, there are no special studies devoted to the cytoskeleton role in the spinal cord neuronal and glial cell changes induced by microgravity.

Most of the spinal cord research results have been obtained in the ‘Bion-M1’ mice experiment. Nevertheless, the ‘Bion-M1’ program also included a complex study of thick-toed geckos (*Chondrodactylus turneri* GRAY, 1864) in the prolonged space flight experiment and it would be interesting to study the changes in the spinal cord of these animals. Reptiles are a rather exotic object for space biology research. However, some of reptilian specimens possess a number of advantages not presented by other model animals [32,33,34]. Thick-toed geckos were successfully used in the research projects onboard the ‘Foton-M2’ (2005), ‘Foton-M3’ (2007), and ‘Bion-M1’ (2013) unmanned spacecrafts. One of the main advantages of geckos is that the subdigital pads on their toes help them maintain an adhesion in weightlessness, which leads to almost normal behaviour and locomotion, helping to reduce stress caused by flotation [32,35,36]. It was assumed that the gecko’s nervous system in weightlessness controls their attachment to the surfaces by means of inhibition of vestibular signals about ‘free fall’. Control of the gecko attachment by the nervous system is confirmed by the fact that quiescent (presumably sleeping) geckos floated in weightlessness 4.5 times more often than active animals [32].

Complex histological and immunohistochemical studies of internal organs revealed only reversible adaptive changes in the geckos of the flight groups. Apparently, geckos do not require vestibular stimulation by circling (running during the space flight was not observed in any of these animals), but perhaps they use head movements for this [37]. In addition, gecko cerebellum was studied after a 30-day space flight onboard ‘Bion-M1’, including immunohistochemical investigation of neuron and astrocyte cytoskeletal antigens—beta-3-tubulin (TUBB3) and glial fibrillar acid protein (GFAP). It was shown that cytoskeleton elements could be involved in alteration of the nervous system under the space flight conditions [34]. All of these changes in the cerebellum were transitional and quickly reversible. It was supposed that abnormal vestibular afferentation was compensated by the tactile stimulation [32,38,39].

In order to confirm this hypothesis, we examined the forelimb toes and cervical part of the spinal cord of the lacertid thick-toed geckos (*Chondrodactylus turneri* GRAY, 1864) after completion of space flights onboard the ‘Foton-M3’ and the ‘Bion-M1’ satellites (Russia, 2007, 2013), using cytoskeleton markers (neurofilament 200 [NF 200], TUBB3, and GFAP).

## 2. Materials and Methods

### 2.1. Animals

The current study was a part of a complex investigation of geckos after the ‘Foton-M3’ (12 days, 14–26 September 2007) and ‘Bion-M1’ (30 days, 19 April–19 May 2013) space missions. Mature, virgin, female thick-toed geckos (*Chondrodactylus turneri* Gray, 1864) aged 1.5–2 years were studied. Information about animals and experimental conditions are contained in [37,35], respectively.

### 2.2. Ethical Statement

#### 2.2.1. ‘Foton-M3’

The work was carried out according to the protocols of the Biomedical Ethics Commission of State Scientific Center of the Russian Federation Institute of Biomedical Problems of the Russian Academy of Sciences (SSC RF–IBMP RAS): no. 204 of 07/19/07. Experimental animals were euthanised with Nembutal, then decapitated according to American Veterinary Medical Association (AVMA) standards [40].

#### 2.2.2. ‘Bion-M1’

The study was approved by the Biomedical Ethics Commission of SSC RF–IBMP RAS (protocol no. 319, 4 April 2013) and was conducted in compliance with the AVMA standards [40]. Euthanasia of geckos was conducted by intraperitoneal injection of Nembutal. The dissection of flight geckos of both ‘Foton-M3’ and ‘Bion-M1’ satellites was performed within 13.5–16.5 h after satellite landing. The dissection of control animals was performed in accordance with the scheme of the experiment.

### 2.3. Histology

In the experiment ‘Foton-M3’, the second and third right forelimb toes of 5 geckos from the flight (F) group and the same amount from synchronous control (SC) group were cut off. All of these samples were fixed in 4% buffered formalin (pH 7.4), decalcified in nitric acid mixture (6%), and embedded in paraffin; then, serial sections (10 μm) were prepared for 5 F and 5 SC geckos. For each group, the toes of 2 animals were sectioned sagittally and the toes of 3 animals were sectioned frontally. In total, toes of 10 geckos were studied. Two series of sections were made from each toe, using every other section. Mallory staining was used to visualise the internal structure of the toe for one series. Another series of sections was used for immunohistochemistry.

In the ‘Foton-M3’ and ‘Bion-M1’ experiments, geckos’ brains (whole) and the cervical section of the spinal cord were removed. All samples were fixed in 4% buffered formalin (pH 7.4) and embedded in paraffin then serial frontal and sagittal sections (10 μm) were prepared. Brain sagittal sections were made for two animals from the F group and for one from the SC groups in the ‘Bion-M1’ experiment. Frontal sections were made for two geckos from the F group and for two from SC groups in the ‘Foton-M3’ experiment, and for five geckos from F group and for four from SC groups in ‘Bion-M1’ experiment. In total, the brains of 16 geckos were studied. Nissl staining of the total neuronal population was carried out. For the Nissl routine histology, series of each 5th section were used in experiment ‘Foton-M3’, and series of each 10th section were used in experiment ‘Bion-M1’.

### 2.4. Immunohistochemistry (IHC)

For immunohistochemical study of gecko brains and toes after completion of space flights, sections were dewaxed, rehydrated, and treated with a 3% solution of H_2_O_2_ to block endogenous peroxidase. Table 1 summarises the primary antibodies and the labelling procedures used in the study of gecko toes and brains.

Reactions were detected with the UltraVision ONE detection system (Thermo Fisher Scientific, Eugene, OR, USA) with diaminobenzidine (DAB) as the chromogen (Thermo Fisher Scientific Inc., Fremont, CA, USA). To identify neural elements in geckos’ toes, we used DAB with NiCl_2_ formed the end product of dark blue colour, which made it possible to distinguish a positive NF reaction from melanocytes, which had colours from light brown to almost black.

Double immunofluorescent labelling to TUBB3 and GFAP was also applied to analyse the distribution of neurons and glial elements in the spinal cord. For immunofluorescent labelling, dewaxed and rehydrated sections were incubated with blocking solution consisting of 10% normal goat serum (Santa Cruz Biotechnology, Santa Cruz, CA, USA) in Tris-buffered saline plus 0.1% Tween 20 (TBST; Thermo Fisher Scientific Inc., Eugene, OR, USA) for 30 min at room temperature. Primary antibodies were diluted in 1% normal goat serum in TBST and incubated at 4 °C for 24 h. The following primary antibodies and dilutions were used: mouse monoclonal antibodies to GFAP (ready to use; RRID:AB_721051, Thermo Fisher Scientific Inc.) and rabbit polyclonal antibodies to TUBB3 (1:50; RRID:AB_297840, Abcam, Cambridge, UK). Secondary antibodies were diluted in TBST and incubated at 37 °C for 2 h. The following secondary antibodies and dilutions were used: AlexaFluor^®^488 goat anti-mouse IgG (H + L) (1:200; RRID:AB_138404, Molecular Probes^®^; Thermo Fisher Scientific Inc., Eugene, OR, USA) and AlexaFluor^®^555 goat anti-rabbit IgG (H + L) (1:200; RRID:AB_141761, Molecular Probes^®^; Thermo Fisher Scientific Inc.). Sections were covered using Ultra Cruz mounting medium containing DAPI (Santa Cruz Biotechnology, Dallas, TX, USA).

Negative control sections, in which the primary antibody was omitted, were used for each case in each immunostaining run. Reactions on samples of adult human brain were used as positive controls.

### 2.5. Microscopy and Morphometric Analysis

Depending on the applied method, sections were analysed with a light microscope AxoiImager A1 (Zeiss, Gottingen, Germany) or DM 2500 (Leica Microsystems, Wetzlar, Germany) equipped with a digital camera (Lomo, Saint Petersburg, Russia) and McrA-View 7.1.1.2 software (Lomo, Saint Petersburg, Russia), or a fluorescent microscope (Micromed 3 LUM LED; Ningbo Sheng Heng Optics & Electronics Co., Ltd., Gao Qiao, Yin, Ningbo, China) equipped with a digital camera and its corresponding software (Toupcam and ToupView 3.7 software; Hangzhou ToupTek Photonics Co., Ltd., Xiyuan, Hangzhou, Zhejiang, China).

In the experiment ‘Foton-M3’, serial sections of the geckos’ toes and cervical spinal cord were studied only on the qualitative level. In the experiment ‘Bion-M1’, quantitative and qualitative analysis on the serial sections of the cervical spine stained using Nissl and IHC were run. Four series (each 10 sections) were examined: the first series was stained according to Nissl, the second—IHC with anti-TUBB3 antibodies, the third—IHC with anti GFAP antibodies, and the fourth—immunofluorescence with anti-TUBB3- and anti-GFAP antibodies. Spinal cord (cervical section) sections applied with cresylviolet according to Nissl or with immunoperoxidase labelling were digitised with Mekos-C2 system (Russia) on the base of AxoiImager1 and kept as Aperio files.

For experimental neuroanatomical studies of the reptilian spinal cord, a detailed subdivision of the grey matter is necessary. The most widely adopted scheme for the subdivision of the spinal grey matters stems from cytoarchitectonic studies in cats [41,42]. The same principles of laminar organisation can be applied to the reptilian spinal cord [43]. The spinal grey matter of the reptiles includes 10 areas [44]. The delineation of cell groups has been performed as in [43] with the help of the usual cytoarchitectonic criteria: (a) the size and shape of the somata, (b) the disposition of the Nissl substance, and (c) the density and pattern of the cell arrangement [43]. Regarding the cell size, four rather arbitrarily chosen categories of cells were distinguished [44]—small cells: 8–19 mkm, medium cells: 20–29 mkm, large cells: 30–40 mkm, and very large cells >40 mkm.

Digital serial sections of the gecko spinal cord were analysed with Aperio Image Scope, Version 12.3.3.5048. On the Nissl-stained sections, motor neurons of the IX zone were calculated. Motor neurons, cut through the nucleus with visible nucleoli, were included in the analysis. Motor neurons with structural abnormalities (hyper- or hypochromatosis, chromatolysis, vacuolisation) to the total number of motor neuron proportion was evaluated. On the adjoining sections applied with anti-TUBB3, immunoreactive cells were calculated. The proportion of TUBB3-immunoreactive motor neurons was calculated as the ratio of their number to the total number of motor neurons taken as 100%. On the serial section applied with anti-GFAP, astrocyte quantity was calculated in the inferior horn. TUBB3-immunopositive motor neurons to astrocyte ratio was also calculated.

### 2.6. Statistical Analysis

A statistical software package was used (Statistica 10.0, Statsoft Inc., Tulsa, OK, USA). Data were compared with Mann–Whitney *U* nonparametric tests to search differences between two groups. The *p*-value < 0.05 was considered statistically significant.

## 3. Results

### 3.1. Mechanoreceptors

**Figure 1:**
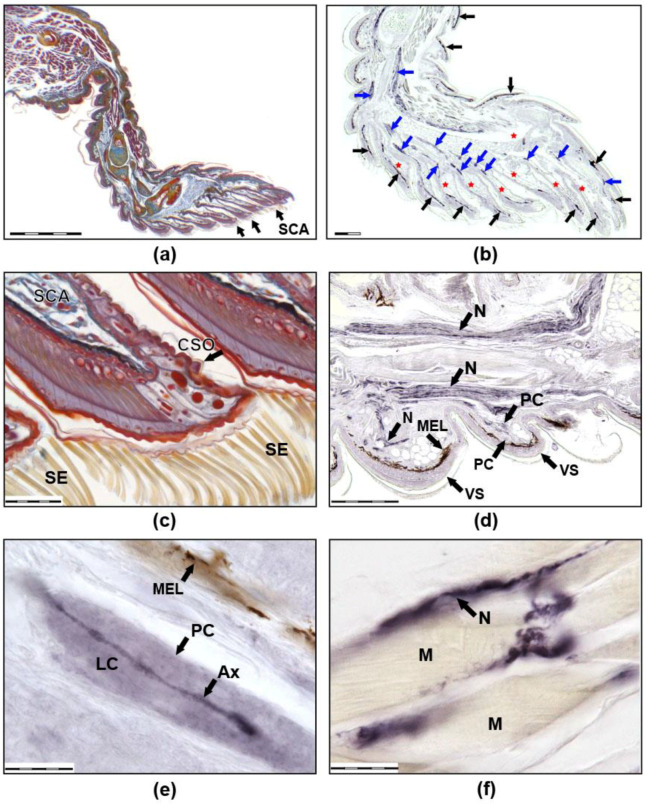
Images (**a**,**b**) show sagittal sections of the third right anterior toe of a thick-toed gecko from SC group. (**a**) Mallory staining: the muscles are burgundy, the connective tissue is blue, the cartilage is yellow-blue, the bone is red, and the nerves are red-blue. (**b**) Immunohistochemical reaction with antibodies to NF 200. Positively coloured structures appear dark blue due to the use of NiCl_2_. Melanocytes look black-brown (not stained). Black arrows show melanocytes, blue arrows show nerves. Venous sinuses and their branches in the scansors are marked with red asterisks. (**c**) Distal parts of scansors, Mallory staining. Numerous setae on the ventral side of the scansor and CSO on the dorsal side of the scansor are visible. Images (**d**–**f**) show the immunohistochemical reaction with antibodies to NF 200 at higher magnification. (**d**) Large nerves are visible in the third phalanx of the toe and thinner nerves that innervate the ventral scales. (**e**) A single PC. (**f**) Muscle innervation. SCA: scansors or subdigital lamellae, SE: setae, CSO: cutaneous sense organ, VS: ventral scales, N: nerves, MEL: melanocytes, PC: Paciniform corpuscle, Ax: axone, LC: lamellar capsule, M: muscles). The size of the scale bars are as follows: (**a**) 500 mkm, (**b**,**d**) 200 mkm, (**c**) 50 mkm, and (**e**,**f**) 20 mkm.

**Figure 2:**
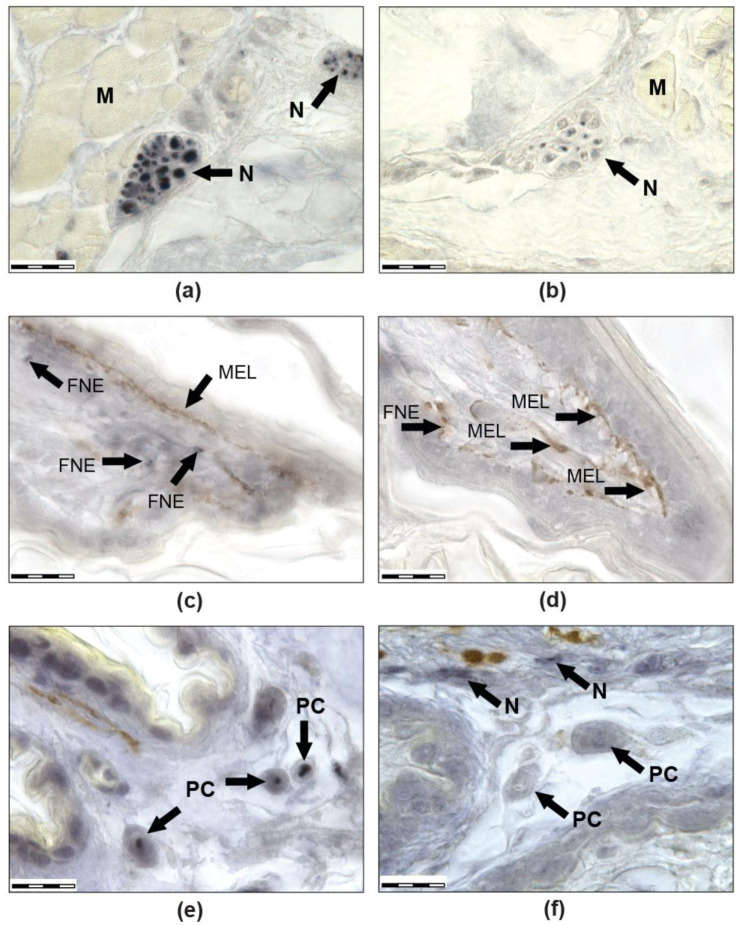
Transversal secctions of the third right anterior toe of a thick-toed gecko, immunohistochemical reaction with antibodies to NF 200. Images in the left column (**a**,**c**,**e**) show the delayed synchronous control, while images in the right column (**b**,**d**,**f**) show geckos after a 12 day spaceflight. Images (**a**,**b**) show the cross sections of large nerves. It can be seen that there were significantly more immunopositive nerve fibres in the control group (**a**) than in the flight group (**b**). Images (**c**,**d**) show the dorsal scales of the third phalanx of the toe; at least 3 free nerve endings are visible in the scale of the control gecko (**c**) and only one is possibly visualised in the flight gecko (**d**). Images (**e**,**f**) show PCs on the cross sections. In the control gecko (**e**), a positively stained central fibre can be seen in each PC; it is not visualised in the flight gecko (**f**). M: muscles, N: nerves, MEL: melanocytes, FNE: free nerve endings, PC: Paciniform corpuscle. The scale bars represent 20 mkm.

In the ‘Foton-M3′ experiment, abundant toe innervation and multiple sensory endings were detected in both groups (Figure 1 and Figure 2). Free nerve endings (FNE) (Figure 2c,d), lamellar or Paciniform corpuscles (PC) (Figure 1d,e and Figure 2e,f), nerve endings (NE) on the clusters of cells with clear cytoplasm (Meissner-like corpuscles, MLC), NE on separate small cells with clear cytoplasm similar to Merkel cells, and NE on separate large cells with granular cytoplasm were found. There were also cutaneous sense organs (CSO), which protrude above the epidermal surface as a cup-shaped structure with a single cilium in the centre (Figure 1c). Most numerous were first two types of mechanoreceptors. In all groups, intraepithelial NE, innervation of muscles (Figure 1f), melanocytes, and blood vessels were observed. Herein is a first description of innervation and variability of receptors in the toes of thick-toed geckos, where PC, FNE, and NE on accessory cells and CSO were found.

All types of mechanoreceptors varied in size, structure, and localisation in both groups. PC is an evolutionary ancestor of the Pacinian corpuscle of mammals including humans. Functionally, they are rapidly adapting low-threshold mechanoreceptors, sensitive to pressure changes and vibration [45]. Variability of PCs in the gecko toe were shown. PCs are typically cudgel-formed, but some of them branched in biparous or a tree-like manner. Sometimes more than one nerve fibre was observed on transverse sections of PC. Transverse sizes of PCs ranged from 7 × 15 to 10 × 23 mkm. PC nerve fibre diameters ranged from 0.5 mkm basement thickness and 2–2.5 mkm apex thickness. Average PC length was calculated as 100 mkm. The maximum PC length of 290 mkm was described in the dorsal part of the phalange.

MLCs can be distinguished by size, structure, and number of included cells. CSOs were revealed in dorsal and ventral scales of phalanges at considerable distance from one another. Their cilia direct forwards to the distal part of the toe. In some cases, CSOs are located on the dorsal side of the scansor, or on its rostral border. On the dorsal side of the scansors, CSOs are found much less frequently than on the dorsal or ventral scales of the phalanges; however, 1–2 CSOs were found in almost all scansors. CSOs located on the scansors are distinguished by a significantly longer cilium length (about 70 mkm) compared to CSOs in scales (15–20 mkm).

Innervation of muscles is extremely variable in both groups. Receptor-like structures of the different size and morphology were described within muscle fascicles (Figure 1f). In all scansors (subdigital lamellae), we observed abundantly innervated ramifications of venous sinuses (Figure 1b), which can take part in adhesion according to literature [46]. The innervation carried out by fibres of two types, 0.1–0.3 mkm and 1–2 mkm, which went parallel to each other and entered through exclusively ventral sinus walls. Small vessels were poorly innervated. Intraepithelial fibres were rarely observed within the scansors and dorsal phalanx parts but are numerous in fold between the proximal phalanx and the palm.

Thus, innervation morphology was generally the same between flight and control groups. Immunoreactivity of the neurofilaments was revealed in both groups. Nevertheless, in the flight group, there were lower or fractionally immunoreactive fibres or immunonegative ones, but these differences should be defined only as a tendency. If the control is dominated by PCs with immunopositive fibres, then in geckos from the flight group, PCs are even more difficult to detect due to the fact that the central fibre is weakly coloured or there is no staining at all. Another difference was revealed in PC morphology: the PC capsule from the control group had well-defined laminar structure, while the PC capsule structure from the flight group was unclear (laminated structure was poorly visible). Moreover, we can say that there are fewer free nerve endings in F than in SC. This may have been due to their reduction but is more likely due to the fact that, with a small diameter, they turn out to be almost unstained and indistinguishable in flight animals.

### 3.2. Spinal Cord

At first, the program of the comprehensive study of the state of geckos after the ‘Foton-M3′ flight did not include the study of the spinal cord. Nevertheless, spinal cord serial frontal sections at levels C1–C5.6 of the two geckos from flight and two geckos from synchronous control groups were at our disposal. As a result of the preliminary research, no visible differences between groups were detected.

**Figure 3:**
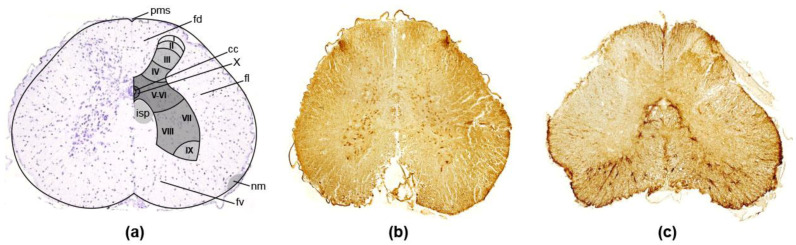
Low magnification of the transverse sections of spinal cord of the thick-toed geckos from the F group showing the general distribution of (**a**) Nissl staining, (**b**) TUBB3 immunoreaction, and (**c**) GFAP expression in the grey and white matter. Pms—posterior median sulcus, cc—canalis centralis, fd—funiculus dorsalis, fl—funiculus lateralis, fv—funiculus ventralis, nm—nucleus marginalis, isp—interstitiospinal tract.

In the ‘Bion-M1’ experiment, in general, the histological appearance of the spinal cord was also normal in all groups of geckos. In the thick-toed geckos (as in several other groups of lacertilians [43,44]), the transverse sections of the cord showed a central, four horned area of grey matter surrounded by an area of white matter (see Figure 3). A deep anterior median fissure and a shallow posterior median sulcus are easily recognised on the spinal cords of all the studied reptiles.

The grey matter of the reptilian cord shows a clear division into ventral and dorsal horns. The dorsal horn receives a dorsal root input; the ventral horn contains the motoneurons. A large intermediate zone remains between the dorsal and ventral horns, which consists mainly of interneurons. The dorsal horns separate off a portion of the white matter, the so-called dorsal funiculus. The remainder of the white matter can be subdivided into lateral and ventral funiculi [43].

#### 3.2.1. Dorsal Horn

In the dorsal horn of the thick-toed geckos, four more or less distinct cellular areas can be recognised, the first three of which show a distinct laminar arrangement (Figure 3a) as in the dorsal horn of *Tupincarbis nigropimatatus* [43]. On the Nissl-stained sections, Area I was shown to consist of a single layer of small round cells, with relatively large, clear nuclei with indistinct nucleoli that caps the dorsal horn. Area II was shown as being directly ventral to area I and following its contours. It consisted of small, rather loosely arranged cells. Most were spindle-shaped with round, clear nuclei and sparse Nissl substance. Area III was an almost cell free zone, lying ventral to area II. Area IV was shown to constitute the main part of the dorsal horn. Area IV was a zone of very tightly packed, spindle-shaped, medium-sized cells with round, clear nuclei and dark nucleoli. Areas V–VI were seen as a relatively wide zone that can be clearly distinguished from area IV due to distinct differences in cytoarchitecture. In thick-toed gecko, such as in *Tupinambis* [43], areas V–VI can be divided into lateral and medial zones. Histopathological observations showed that areas I–VI of the spinal cord of geckos had a normal morphological appearance in both groups. There was no sign of chromatolysis or shrinkage of neurons. In general, neurons in these regions were neatly arranged with an apparent nucleus, and the cytoplasm had a normal distribution of Nissl bodies. Immunoreactivity patterns found by using markers were generally uniform in both groups.

#### 3.2.2. Ventral Horn

Areas VII–VIII contained numerous medium-sized polygonal cells (20–30 μm) with round and distinct nucleoli. Large neurons with a fine granular Nissl substance were found, particularly in the medial ventral horn. Round to oval, small cells with round nuclei and distinct nucleoli occurred. In *Chondrodactylus turneri,* such as in *Tupinambis* [43], at least in the enlargements, the area can be divided into a dorsolateral (area VII) and a ventromedial (area VIII) zone.

**Figure 4:**
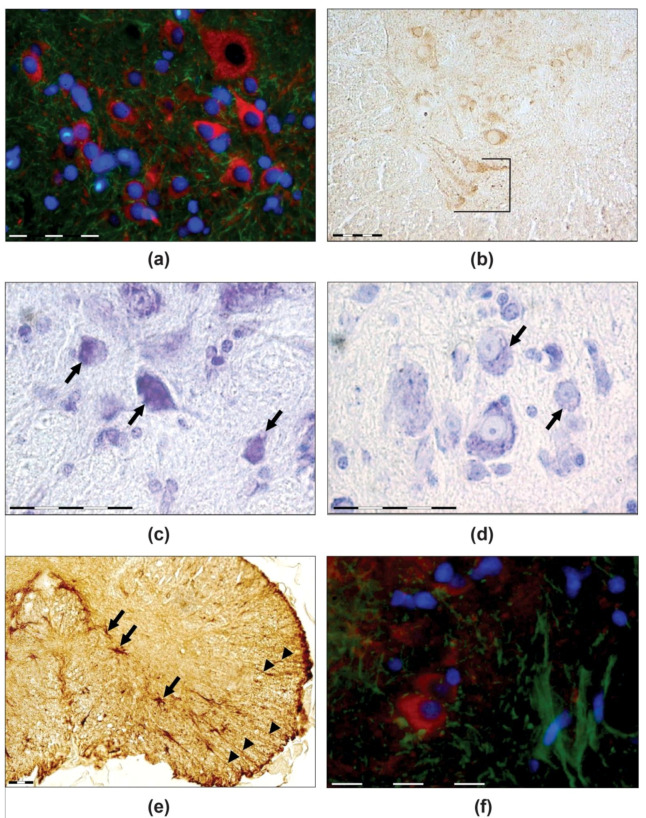
(**a**) TUBB3 (red) and GFAP (green)-immunoreactive cells of the ventral horns of geckos’ spinal cord in control. (**b**) A palisade-like arrangement of motor neurons in the cervical enlargements in a gecko after spaceflight, TUBB3 immunoreaction. (**c**) Hyperchromic motor neuron with vacuolisation in a gecko’s spinal cord after spaceflight, Nissl staining. (**d**) Normochromic motor neurons with vacuolisation (control group), Nissl staining. (**e**,**f**) Star-shaped astrocytes (arrows) in the lower part of the spinal cord ventral horn. Some radial glial cells were displaced laterally (arrowheads). Image € shows the flight group (GFAP immunoreaction), while image (**f**) shows the control group (immunofluorescence: red-motor neurons (TUBB3), green-star-shaped astrocytes (GFAP), blue-cell nuclei (DAPI). Scale bars—50 mkm.

Area IX, i.e., the motoneuron area (Figure 3 and Figure 4a,b), consisted of two longitudinal columns—medial and lateral ones. The medial column, which was presented throughout the spinal cord, is related to the innervation of the neck, trunk, and tail musculature [44]. The lateral column was presented only in the cervical enlargements and is related to the innervation of the extremity muscles. In thick-toed geckos, the cells of the lateral column showed a palisade-like arrangement.

Area X, which surrounds the central canal, can be distinguished throughout the spinal cord. This area, also called substantia grisea centralis, was composed of very small (4–6 mkm), round cells with round nuclei and indistinct nucleoli. In addition, the *marginal* or *edge cells*, demonstrated in a wide variety of reptiles [44], were laying just beneath the pial surface and dorsal to the ventral roots.

Nonspecific heterogenetic changes, such as chromatolysis, vacuolisation, and hyperchromatosis (Figure 4c,d), were observed in the IX area of geckos from the flight group. Chromatolysis of the basophilic substance of the cytoplasm (tigroid) was observed in varying degrees. However, segmental chromatolysis was extremely rare. Moreover, there were no significant differences in relative quantity of motor neurons with such pathomorphological changes to all motor neurons between F and SC groups (*p* > 0.05). In addition, the relative number of the TUBB3-immunopositive motor neurons was the same in both groups (*p* > 0.05).

Clear GFAP immunopositivity with thin, radial-oriented immunopositive processes evident throughout the cross-sectioned spinal cord of thick-toed gecko (Figure 3c and Figure 4e,f), as in *Podarcis* and *Anolis sagrei* [47,48], was shown. The spinal cord ependyma exhibited no GFAP staining. Radial astrocytes were characterised by cell bodies displaced away from the ependymal layer into a periependymal position, preferentially aggregated in the dorsal and lateral part of the grey matter (Figure 3c). They gave origin to the radial processes directed towards the spinal cord surface.

In the spinal cord, star-shaped astrocytes coexisted with radial glia. Star-shaped astrocytes were evident in the ventral horns at the boundary between white and grey matter (Figure 4e,f). No signs of gliosis or changes in radial glial distribution were observed. No differences in the TUBB3-immunopositive motor neurons to star-shaped astrocyte ratio were identified between the two groups (*p* > 0.05).

## 4. Discussion

Nervous system integration is necessary for movement control, sensory navigation, and terrestrial locomotion of Earth species, including humans. It maintains muscle contraction, allowing animals to react against gravitational force and controlling motions. One of the most fundamental nervous circuits is a classic reflex arch. Spinal reflexes are the simple neuromuscular reaction of proportional magnitude to the input stimulus. In addition, sensory inputs from the vestibular, visual systems, and proprioception integrate by the central nervous system (CNS) to control posture and movement [16].

Geckos are mainly small insectivorous lizards of warm climates. Their ability to scale walls and to run upside down across ceilings depends partly on the presence in most species of extremely sharp recurved claws: geckos rely on their claws to maintain purchase on rough, steeply inclined surfaces [49]. However, in some gecko species, this ability is mainly based on the structure and function of adhesive subdigital pads on the underside of the toes [50]. These pads consist of a series of modified lamellae (scansors) [51], covered with uniform arrays of similarly oriented hair-like bristles (setae) [52,53,54]. Climbing adaptations of very different types and in different conditions are found among the geckos [55,56,57,58].

Thus, on the one hand, there is an extensive literature on adhesion mechanisms in geckos. On the other hand, structure and function of reptile cutaneous mechanoreceptors were deeply investigated using electrophysiological, morphological, histological, and electron-microscopical methods in the years 1960–1970 and beyond [59,60,61,62,63,64,65,66]. At the same time, relatively few works have been written on the innervation and mechanoreceptors of the gecko’s toes, and they are mainly devoted to cutaneous sensilla (skin sensory organs) [67,68,69,70,71]. Thus, the role of the nervous system in geckos’ adhesion remains deficiently investigated.

NF 200 is proposed as the most useful marker for the mechanoreceptors and other neuronal structures of the thick-toed gecko research after a series of immunohistochemical experiments with a wide marker panel: antibodies to protein S-100, NSE, neurofilaments (Sigma, St. Louis, MO, USA, Cell Marque, Rocklin, CA, USA, LabVision, Bucharest, Romania), PGP9.5, neuron-specific beta-III-tubulin, GAP-43, NCAM, SNAP-25, synaptophysin, and G-proteins were tested. In most cases, there was no positive reaction, or an extremely weak specific staining was observed. Maybe it was because previous decalcification of gecko toes. Clear positive reactions were obtained only with antibodies to NF 200.

Several new points of toe innervation patterns were revealed with anti-NF 200 in the geckos. Various types of mechanoreceptors have been discovered, and CSO on scansors of thick-toed geckos has been described. It is known that CSOs have not only mehanoreceptive functions but they also may be potentially involved with additional sensory modalities. At the same time, their location on the scales’ and scansors’ margins and the direction of their bristles to adjacent scales shows that CSOs are important for proprioreception during toepad deployment [71].

Differential NF 200-immunoreactivity and unstained areas in the nerve fibres were observed in both groups; however, more low- or partly immunoreactive or completely immunonegative nerve fibres were revealed in the flight group. Fewer free nerve endings were detected in the F group than in the SC one. In the gecko PCs of the F group, the layered structure of the capsule was not visible or weakly expressed, and the central fibre was immunonegative or partially immunoreactive and much thinner or hypertrophied and empty, in contrast with geckos from the SC group.

Thus, even after a short-term 12 day space flight, some changes in the nervous system of the toes were observed. NF 200-immunoreactivity pattern alteration could be led from destruction, synthesis changes, and/or rearrangement of neurofibrillae. According to video records, geckos from F group retained attachment to the walls of the container in weightlessness and their behaviour did not differ significantly from SC. Further investigation is needed to clarify mechanisms and functional value of the changes revealed in mechanoreceptors of gecko toes. The role of nervous system in geckos’ adhesion and locomotion call for further space and terrestrial studies.

Spinal cord changes with increasing complexity of animal locomotion—terrestrial quadruped animals have cervical and lumbar enlargements. However, relatively limited data are available on the physiology of the reptilian CNS. Thus, any functional correlations with mammal spinal cord should be made very carefully. Since area IX plays a leading role in the formation of movements, we conducted a study of the cytoskeleton of motor neurons in this area. Area IX is a primary motor area with somatotopic organisation. In quadrupedal reptiles, in addition to a medial column related to the neck, trunk, and tail musculature innervation, a lateral column is added in the area of cervical and lumbosacral enlargements. The motoneurons of lateral column innervate the limb muscles [44].

Motoneuron swelling, central and segmental chromatolysis, and hyperchromatosis were revealed in flight group geckos. These morphological alterations are nonspecific and possessed under different nonpathological statements, including functional loading and pathogenetic disturbances, such as trauma, ischemia, intoxication, and chronic stress. Such changes revealed in the motor neurons of the flight group geckos were insignificant in comparison with control group. There were also no changes in the TUBB3 immunoreactivity distribution.

However, in many studies of immunoexpression in mice spinal cord in the ′Bion-M1′ program, the expression of some proteins was decreased. Thus, Porseva et al. [17,72] showed that the number of neurons containing ChAT and neurofilament proteins decreased in the thoracic section of the spinal cord in mice after the 30 day spaceflight. Moreover, comparative analysis of transcriptome revealed gene expression changes after 30 days of a HUM experimental study. The decreasing of 37 gene expressions, including genes related to cytoskeleton and cell adhesion (VIM, DRP2, CLDN19, CD9, SPON2, SMTN), were described [13]. In addition, the immunohistochemical assay was also used to investigate reactions of mice lumbar motor neurons after the ‘Bion-M1’ space flight [73]. The decreased immunoexpression of synaptic proteins (synaptophysin and postsynaptic density protein 95) in motor neurons of mice after the spaceflight is consistent with more than 15-fold upregulation of the corresponding genes (*Syp* and *Dlg4*). Both microgravity and the simulation of microgravity induced structural changes in the large neurons of lumbar spinal ganglia and motoneurons of the lumbar spinal cord [14].

At the same time, the number of calbindin-positive neurons increased; motoneurons, expressing neuronal NO synthase and caspase 3 appeared, while Ki-67 was not detected in the spinal cord of the mice after the ‘Bion-M1’ space flight. Cytoplasmic vacuolisation was seen in motoneuron staining with thionine and immunoreactive for calbindin [17,72]. According to the results of Kuznetsov, in a week after landing, the level of ChAT gene revealed a 17-fold increase; levels of Nefl, Nefm, and Nefh genes increased 176, 284, and 176 times, respectively. Changes in the level of proteins in motoneurons resulted, possibly, in increased expression of the gravity-sensitive genes during the readaptation period [8].

In general, morphological and immunohistochemical changes in motoneurons reflect adaptive mechanisms to the conditions of space flight [17].

Space flight influence on the glial cell populations is poorly studied. GFAP is an intermediate filament protein of certain glial cells—typical star-shaped fibrous or protoplasmic astrocytes, Bergmann glia, periependymal radial glia, and tanycytes. GFAP is a stable protein by its molecular and antigenic characteristics throughout vertebrate species [47,48]. The present immunocytochemical study revealed that the GFAP immunoreactivity of geckos CNS is intense. This is in accordance with studies performed on other reptiles [47,48,74].

Neuron–astrocyte system alterations influence neuronal structure and function up to stable motoneuron disturbances, leading to cell degeneration and death. Astrocytes specifically react on the damaging factors for nervous system protection and recovery. Increasing of the astrocyte perikaryon and ramification, activation of the protein synthesis, including S100B, cytoskeleton antigens such as glial fibrillary acidic protein (GFAP), vimentin, and nestin appear due to stress or damaged factors [75]. Increasing of astrocyte quantity—astrogliosis—is agreed as a sign of neurodegeneration [75].

The pattern of the glial fibrillary acidic protein (GFAP)-immunopositive staining in the gecko brain was also studied in the ‘Foton-M2’ and ‘Foton-M3’ experiments. Immunoreactivity in the medial and media-dorsal cortex was lower in the brain of flight group geckos after ‘Foton-M2’ and ‘Foton-M3’ than in the brain of control group geckos in these experiments [33]. The findings coincide with the data on GFAP expression level decreases in the rat hippocampus after microgravity [15]. At the same time, no differences were found in the immunoreactivity patterns and intensity of reaction with GFAP antibodies in the cerebellum between the flight and control groups in geckos after the completion of spaceflights on board ‘Bion-M1’ [34]. In our study, no alteration or disturbances of the geckos’ spinal cord astrocyte population were also revealed. No signs of gliosis were detected in animals of the flight groups. The absence of visible changes in glial cells of the spinal cord after the spaceflight confirmed the thesis that geckos’ neurons did not undergo critical changes under spaceflight conditions. After completion of the ‘Bion-M1’ mission, the absence of proliferating gliocytes and the persistence of the nucleolar component were also observed in mice spinal cords. It provides evidence of the absence of the necrobiotic changes typical of necrosis [17,72].

At the same time, severe functional impairment of postural and locomotor musculature was obtained in mice after the 30 day spaceflight on board the ‘Bion-M1’ biosatellite [8,76]. Motor function impairment was revealed during behavioural analysis of mice directly at the landing site in the ‘Bion-M1’ experiment. Problems with maintaining steady posture, changing paw positions, denying moving, and other alterations were observed. Some recovery of locomotor function was reported 6–8 h after landing [76].

However, no locomotion disturbances were revealed in geckos in the experiments. In our previous studies, we focused on the prolonged spaceflight effects on the cerebellum [33,34] and concluded that in the CNS, inputs from the vestibular system are compared with visual and proprioceptive signals to control motions and compensate for head and eye movements. In humans, changes in motor inputs descending from the brain are supposed to induce inhibition of spinal reflexes under microgravity and contradictorily, facilitated under hypergravity. It is speculated that vestibular, visual, and somatosensory inputs change under altered gravity, which reduce vestibulosomatosensory answers, leading to vision predominance in microgravity [16].

Otherwise, supporting inputs play a key role in the postural-tonic system regulation. Gravitational loading influences both electric activity and transversal stiffness of muscle fibres. The initial point of the atony development is slow motor unit inactivation, leading to consecutive sarcomere protein destruction and actin–myosin interaction reduction [7]. Seven day exposure to dry immersion caused a 40% decrease of cytoskeleton proteins such as titin, nebulin [77], desmin, and a-actin-2 [78] in humans. At the same time, postural muscle demonstrated no transversal square decreasing and changes in fibres expressed slow/fast myosin ratio after 7 day immersion with supporting stimulation [77]. Atrophy development was negated without intensive running or special resistance trainings [10]. According to these data, it was hypothesised that the supporting afferentation is a key factor in the maintaining of the dilatory tonic motoneuron activity. Compensation of the supporting input absence with the mechanic feet stimulation causes a decrease in functional and structural changes of the muscle fibres induced by dry immersion [77,79,80,81]. It is speculated that contraction activity, induced by the supporting afferents activation, contradicts cytoskeletal chain disintegration, which protects against atrophy [10].

**Figure 5:**
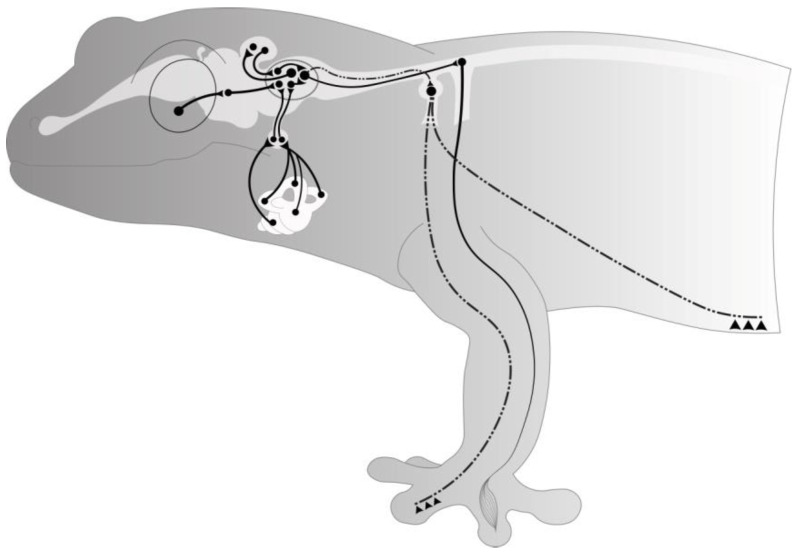
Diagram summarising the vestibular, visual, and somatosensory inputs in *Chondrodactylus turneri* Gray and illustrating the mechanism of the postural-tonic system regulation.

The study results provide evidence that proprioceptive signals may compensate altered sensory afferentation and negate the HMS effects (Figure 5).

These data are also supported by the human experimental results. It has been shown that pressure on the feet of astronauts with the help of special shoes enhances neuromuscular activation and modifies its phasic features when lifting the arm [82]. The pressure on the arms also provides greater accuracy in space flight. In addition, underwater divers are known to reduce alternobaric vertigo, a rotational illusion, by gripping a rock or other stationary object [83]. At the same time, astronauts were shown to have cytoskeleton alteration after prolonged space missions [84].

Recently, zu Eulenburg et al. [84] showed that astronauts, after a long space flight, have signs of disorders of the nervous system (and in particular cytoskeletal disorders). NF levels in the blood samples of cosmonauts was significantly elevated compared with pre-flight levels directly postflight, 1 week, and 3 weeks after return to Earth. GFAP showed a significant increase at the end of the first week postflight and beyond. An increase of tau and amyloid proteins was also detected. They can be of clinical importance. Thus, one of the possible explanations for the mechanisms of Alzheimer’s disease is the tau hypothesis, according to which the strands of hyperphosphorylated tau protein begin to unite with each other, eventually forming neurofibrillary tangles inside nerve cells [85]. This causes the loss of cytoskeletal microtubules and tubulin-associated proteins [86] and the collapse of the transport system inside the neuron [87], leading first to a violation of biochemical signal transmission between cells then to the death of the cells themselves [88]. Similar processes can occur after head trauma, leading to the development of chronic traumatic encephalopathy [89], as well as in some of other age-associated degenerative disorders both in human and animals [90].

## 5. Conclusions

Sensorimotor impairments occurring under the altered gravity limit a space mission duration. Sensorimotor function plays a crucial role in maintenance during space flight and after landing, including lengthy recovery or rehabilitation after returning to Earth [8,11,16].

Some neuromuscular changes in astronauts are closer to the certain pathognomonic patterns observed in skeletal muscles in patients with spinal or peripheral nerve disorders [4]. The certain changes observed in physiotherapy practice on Earth in patients with low back pain, muscle wasting diseases, and exposure to prolonged bed rest. Elite athletes and patients in intensive care closely resemble physiological changes seen in astronauts [4]. According to physiotherapy experience obtained with patients on Earth and astronauts during space missions, regular physical exercises are postulated as the best way to maintain an astronaut’s capacity for work in flight and to prepare them for returning to Earth [6]. The special set of exercises prevent astronauts from developing HMS [91]. The medical practice developed by space medicine may contribute a usefulness for motor dysfunctions research and therapy, such as spinal cord injury and stroke [11].

Our results confirm the compensatory theory (for a review, see [3]) and the tonic-supporting theory of I.B. Kozlovskaya [7]. Complex study of the spinal cord and peripheral mechanoreceptors is necessary for understanding the influence of space flight conditions on Earth organisms. As the spinal cord is an evolutionarily stable structure, thick-toed geckos may be a useful model for space flight studies on the vertebrate spinal cord. The provided methodology approach allows us to reveal the main cell types and cellular changes. According to our research experience, cytoskeleton markers adequately reflect changes in the cells of the nervous system and can be used to study their morpho-functional state. Moreover, our approaches have the potential to further clarify if changes of cytoskeleton in structures of nervous system are gravity-dependent and influence motor control.

## Figures and Tables

**Table 1:** Panel of antibodies used.

Antibody Specificity	Origin	Dilution	Incubation	Source
GFAP	Mouse	Ready to use	60 min, RT	Thermo Fisher Scientific
NF 200	Rabbit	1:160	30 min, RT	Sigma
TUBB3	Rabbit	1:1000	60 min, RT	Abcam

GFAP—glial fibrillar acidic protein; NF 200—neurofilament 200; TUBB3—neuronspecific βIII-tubulin; RT—room temperature.

## Data Availability

The data presented in this study are available on request from the corresponding author.
